# Berberine Ameliorates Chronic Kidney Injury Caused by Atherosclerotic Renovascular Disease through the Suppression of NFκB Signaling Pathway in Rats

**DOI:** 10.1371/journal.pone.0059794

**Published:** 2013-03-26

**Authors:** Xin Wan, Xin Chen, Lin Liu, Ye Zhao, Wen-Juan Huang, Qian Zhang, Gang-Gang Miao, Wen Chen, Hong-Guang Xie, Chang-Chun Cao

**Affiliations:** 1 Division of Nephrology, Department of Medicine, Nanjing First Hospital, Nanjing Medical University, Nanjing, China; 2 Division of Cardiothoracic Surgery, Department of Surgery, Nanjing First Hospital, Nanjing Medical University, Nanjing, China; 3 Division of Nephrology, Department of Medicine, Xuzhou Medical College, Xuzhou, China; 4 General Clinical Research Center, Nanjing First Hospital, Nanjing Medical University, Nanjing, China; 5 Department of Pharmacology, Nanjing Medical University School of Pharmacy, Nanjing, China; INSERM, France

## Abstract

**Background and objectives:**

Impaired renal function in atherosclerotic renovascular disease (ARD) may be the result of crosstalk between atherosclerotic renovascular stenosis and amplified oxidative stress, inflammation and fibrosis. Berberine (BBR) regulates cholesterol metabolism and exerts antioxidant effects. Accordingly, we hypothesized that BBR treatment may ameliorate ARD-induced kidney injury through its cholesterol-lowering effect and also suppression of the pathways involved in oxidative stress, inflammation and NFκB activation.

**Methods:**

Male rats were subjected to unilateral renal artery stenosis with silver-irritant coil, and then fed with 12-week hypercholesterolemic diet. Rats with renal artery stenosis were randomly assigned to two groups (n = 6 each) – ARD, or ARD+BBR – according to diet alone or in combination with BBR. Similarly, age-matched rats underwent sham operation and were also fed with hypercholesterolemic diet alone or in combination with BBR as two corresponding controls. Single-kidney hemodynamic metrics were measured in vivo with Doppler ultrasound to determine renal artery flow. The metrics reflecting hyperlipidemia, oxidative stress, renal structure and function, inflammation and NFκB activation were measured, respectively.

**Results:**

Compared with control rats, ARD rats had a significant increase in urinary albumin, plasma cholesterol, LDL and thiobarbituric acid reactive substances (TBARS) and a significant decrease in SOD activity. When exposed to 12-week BBR, ARD rats had significantly lower levels in blood pressure, LDL, urinary albumin, and TBARS. In addition, there were significantly lower expression levels of iNOS and TGF-β in the ARD+BBR group than in the ARD group, with attenuated NFκB-DNA binding activity and down-regulated protein levels of subunits p65 and p50 as well as IKKβ.

**Conclusions:**

We conclude that BBR can improve hypercholesterolemia and redox status in the kidney, eventually ameliorating chronic renal injury in rats with ARD, and that BBR can act against proinflammatory and profibrotic responses through suppression of the NFκB signaling pathway.

## Introduction

Chronic kidney injury caused by renovascular diseases would be increased over time in patients with end-stage renal disease (ESRD) [1]. Renal artery stenosis, most commonly due to atherosclerotic plaques and atherosclerosis, is an important clinical entity that can lead to hypertension and progressive renal damage [1]–[2]. In addition to threatening renal function, atherosclerotic renovascular disease (ARD) with renal failure poses a risk for exacerbation of cardiovascular disease and predicts cardiovascular mortality [3]–[4]. Hence, the mechanisms responsible for renal damage in this disease are being vigorously sought, and effective therapeutic strategies to preserving the kidney are under intense investigation.

Berberine (BBR), a kind of isoquinoline alkaloid with multiple pharmacological actions, has been widely used as a therapeutic agent indicated for tumor and microbial infection in China and other East Asian countries [5]. In addition, there have been reports showing its potential to treat diabetes and cardiovascular disease [5]–[7]. Evidence has demonstrated that BBR could effectively regulate cholesterol metabolism, inhibit cell proliferation and act against oxidative stress properties [5]–[9]. In this report, we investigated whether BBR could protect against chronic renal injury in the rat models with hyperlipidemia and unilateral renal artery stenosis.

## Materials and Methods

### Animals and experimental design

Normotensive male Wistar rats, weighing 200–220 g, were provided by the Experimental Animal Center affiliated to Nanjing Medical University, Jiangsu, China. Prior to the initiation of experimental protocols, rats were housed with free access to tap water and food for over 1 week. All procedures were performed under sterile conditions in accordance to the guidelines set by the Institutional Animal Care and Use Committee, Nanjing First Hospital, Nanjing Medical University, and the local law on animal care and protection.

In order to make rat model with hyperlipidemia and unilateral renal artery stenosis as required, rats were anesthetized with pentobarbital sodium (40 mg/kg, ip). After that, via a flank incision, a 0.3 mm-diameter silver-irritant coil was placed in the left renal artery at baseline to chronically reduce perfusion pressure, and the right nephrectomy was performed [10]–[11]. Rats were fed with a 12-week hypercholesterolemic diet of 2% cholesterol and 15% lard diet [10]–[12]. Single-kidney hemodynamic measures were undertaken in vivo with Doppler probes (Visual Sonics Vevo 2100 MS-250, Canada) to determine left renal artery blood flow. Rats with renal artery stenosis were randomly assigned to two groups (n = 6 each) – ARD rats with or without administration of BBR (Sigma, St Louis, MO, USA) 150 mg/kg per day by gastric gavage, followed by a 12-week hypercholesterolemic diet to feed [5]–[6]. Similarly, age-matched rats underwent sham operation and were fed with normal diet in the absence or presence of BBR as two corresponding control (CTL) groups – CTL or CTL+BBR. Body weight and food intake were recorded daily during the experimental period.

At the end of 12-week treatment period, rats were placed in individual metabolic cages for a 24-hour urine collection and measurement of food and water intake. They were then anesthetized with pentobarbital sodium (40 mg/kg, ip) and euthanized by exsanguination using cardiac puncture. Blood samples drawn from the inferior vena before PBS perfusion were centrifuged at 3,000 g for 5 min, and their supernatants were stored with the equal volume at −80°C. Urine was collected for measurement of urinary albumin (uALB). After the kidney was harvested, a specimen was fixed, in turn, with 10% formalin and 4% paraformaldehyde, another small fraction was immediately fixed with 4% glutaraldehyde for electron microscopy, and the remaining was cleaned with PBS, snap-frozen in liquid nitrogen, and stored at −80°C until processed [13].

### Measurement of systolic blood pressure

Blood pressure was noninvasively measured by a volume pressure recording sensor and an occlusion tail-cuff with Powerlab/8SP data acquisition system (AD Instruments, AD Instrument, Castle Hill, Australia) as described elsewhere [14]. In brief, rats were trained by placing them in restraints, 1 hour daily, for 7 days before the experiments. Upon completion of the training, conscious rats were restrained, gently warmed using a heating lamp, and took a rest for 10–15 min. The cuff was then placed around the tail, inflated and released several times. After stabilization, systolic blood pressure (SBP) was recorded every week, three times at a time, and an average of recorded values was calculated.

### Measurement of reactive oxygen species (ROS) and antioxidant capacity

Chemiluminescence (CL) was used to measure ROS as described elsewhere [10], [12]. Briefly, renal tissues were incubated in 0.9% NaCl solution containing 10 mM phosphate buffer (pH 7.4), 6 mM KCl and 6 mM MgCl_2_ with the CL probe (Jiangcheng Chemical Company, Nanjing, China). During the incubation period, CL intensity was recorded continuously for 30 min using a Luminescence Reader apparatus [10]. Thyobarbituric acid reactive substances (TBARS), superoxide dismutase (SOD), and catalase (CAT) in kidney cortex were measured, respectively, using commercially available kits (Jiangcheng Chemical Company, Nanjing, China) according to the manufacturer's instructions [12].

### Histological examination

Formalin-fixed tissue fractions (stained with hematoxylin and eosin and Masson trichrome) were evaluated and scored with light microscopy by the same well-trained staff blind to the study assignment. In brief, the degree of tubulointerstitial injury, defined as tubular dilatation and/or atrophy, cell infiltrate or cellular edema, was estimated semi-quantitatively according to the pre-specified following criteria: grade 0: normal kidney; grade 1: damaged up to 25% of the cortex; grade 2: damaged 26 to 50% of the cortex; and grade 3: extensive damage of>50% of the cortex [14]–[15]. Interstitial fibrosis was measured by the presence of interstitial collagen in sections stained with Masson trichrome.

### Immunohistochemical staining

Immunostaining was processed in 3 µm paraffinized sections. Slides were dewaxed, and sections were washed three times for 5 min each in PBST (PBS, pH 7.4, 0.05% Tween 20). Then, the microwave antigen retrieval procedure (citrate buffer, pH 6.0) was performed. Rabbit-anti-rat iNOS or TGF-β (1∶100, Santa Cruz Biotechnology, CA, USA) was used as primary antibodies and incubated overnight at 4°C. Nonspecific binding sites were blocked with 4% goat serum diluted 1∶10 in PBST. The primary antibody was detected by horseradish peroxidase (HRP)-conjugated anti-rabbit/anti-rat secondary antibody (Keygen, Nanjing, China), and developed with DAB chemical kit (Zhongshan Goldbridge, Nanjing, China). Nuclei were counterstained with hematoxylin. All slides were prepared in duplicate, one served as a control for secondary antibody binding specificity. The positive areas were measured with five randomly chosen fields [12]–[13], [15].

### Western blot analysis

The total protein was extracted from rat kidney cortical tissues with ice-cold lysis buffer containing proteinase inhibitor and phosphatase inhibitors (Keygen, Nanjing, China). Protein concentrations were measured using a BCA Protein Assay Kit (Keygen, Nanjing, China). The equal amount of lysate proteins (40 µg) was separated on 15% SDS-PAGE gels (for the detection of iNOS, TGF-β, p-IKKβ, and p65), transblotted onto PVDF membrane, and blocked with 5% skim milk powder in TBST buffer (10 mM Tris, pH 7.5, 150 mM NaCl, and 0.05% Tween 20). Subsequently, the membranes were probed with primary antibody (Santa Cruz Biotechnology), followed by HRP-conjugated second-antibody (1∶5000, Keygen, Nanjing, China). The protein band was detected using the ECL detection system (Keygen, Nanjing, China) [12]–[13], [15].

### Electromobility shift assay (EMSA)

Nuclear proteins from kidney cortical tissues were prepared as described elsewhere [5]. Preparation for EMSA was performed with a gel shift assay kit E3300 according to the manufacturer's instructions (Promega, WI, USA). Briefly, NFκB binding-site DNA was radiolabeled with γ-^32^P and the use of T4 polynucleotide kinase. Nuclear extracts (10 µg) were incubated in binding reaction medium with 0.5 ng of ^32^P-end-labeled oligo, containing the NFκB binding-site DNA for 30 min at ambient temperature. In a competition assay, 50 ng of unlabeled NFκB binding-site DNA or scrambled DNA were used. The DNA-protein complexes were analyzed on 5% polyacrylamide gels, and autoradiographed. Band densitometrics was estimated on autographs and values were expressed as relative density unit (RDU) against control band [5], [13], [15].

### Statistical analysis

All data were expressed as the mean ± SEM. Statistical analyses were performed using ANOVA test, followed by the Bonferroni correction for multiple comparisons. To compare NFκB activities, Wilcoxon rank-sum test was used. The differences were evaluated with SPSS 13.0 software (SPSS, Chicago, IL, USA). At least 3 independent experiments were performed. *P*<0.05 was considered statistically significant.

## Results

### Assessment of the ARD rat model

**Figure 1:**
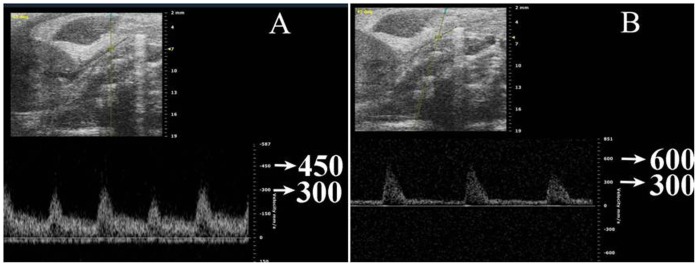
Doppler ultrasound velocity at the left renal artery was detected at 300 mm/sec in CTL rats (A); renal blood flow acceleration was detected at 600 mm/sec in ARD rats (B).

**Figure 2:**
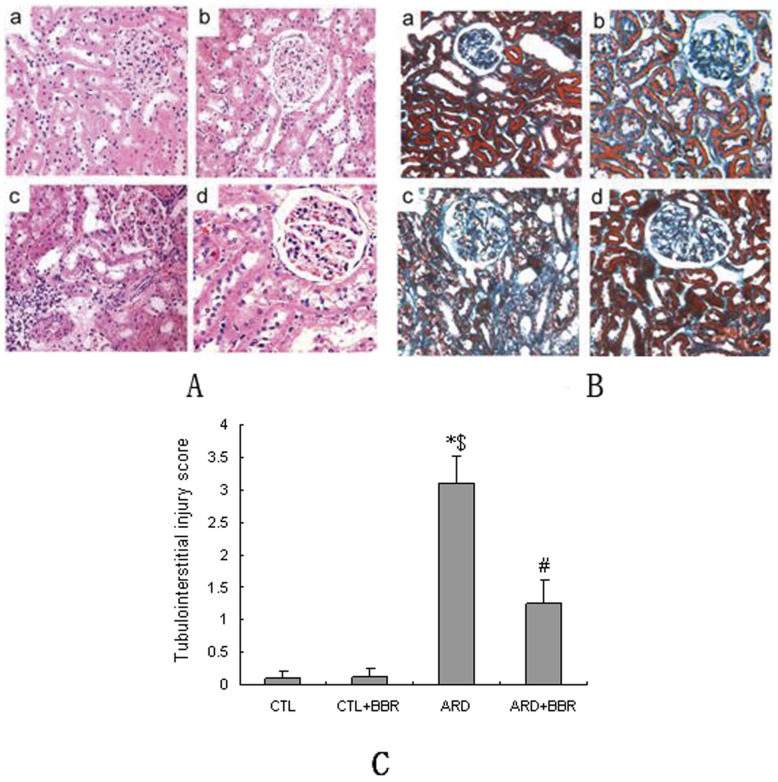
Effects of BBR on renal tissue architecture and fibrosis. **A**: Representative light microscopic features of HE-stained kidney sections. a) Normal histological structure. b) Almost normal histological structure after BBR treatment in CTL+BBR rats. c) Tubulointerstitial inflammation and cells infiltration were evident in ARD rats. d) The renal histological morphology is almost normal after BBR treatment in ARD+BBR rats. **B**: Assessment of kidney tissue architecture, and interstitial and perivascular fibrosis by Masson's trichrome staining. a) Normal histological structure. b) Almost normal histological structure after BBR treatment in CTL+BBR rats. c) Compared with CTL rats, ARD rats had more tubular degeneration and ectasia, more interstitial, periglomerular, and periarterial fibrosis (light blue staining areas). d) The renal histological morphology was almost normal after BBR treatment in ARD+BBR rats. **C:** Quantification of Masson's trichrome for tubulointerstitial injury score. Data expressed as means ± SEM, n = 6 per group. *P<0.05 vs. CTL group. ^#^ *P*<0.05 vs. ARD group. ^$^ *P*<0.05 vs. CTL+BBR

**Figure 3:**
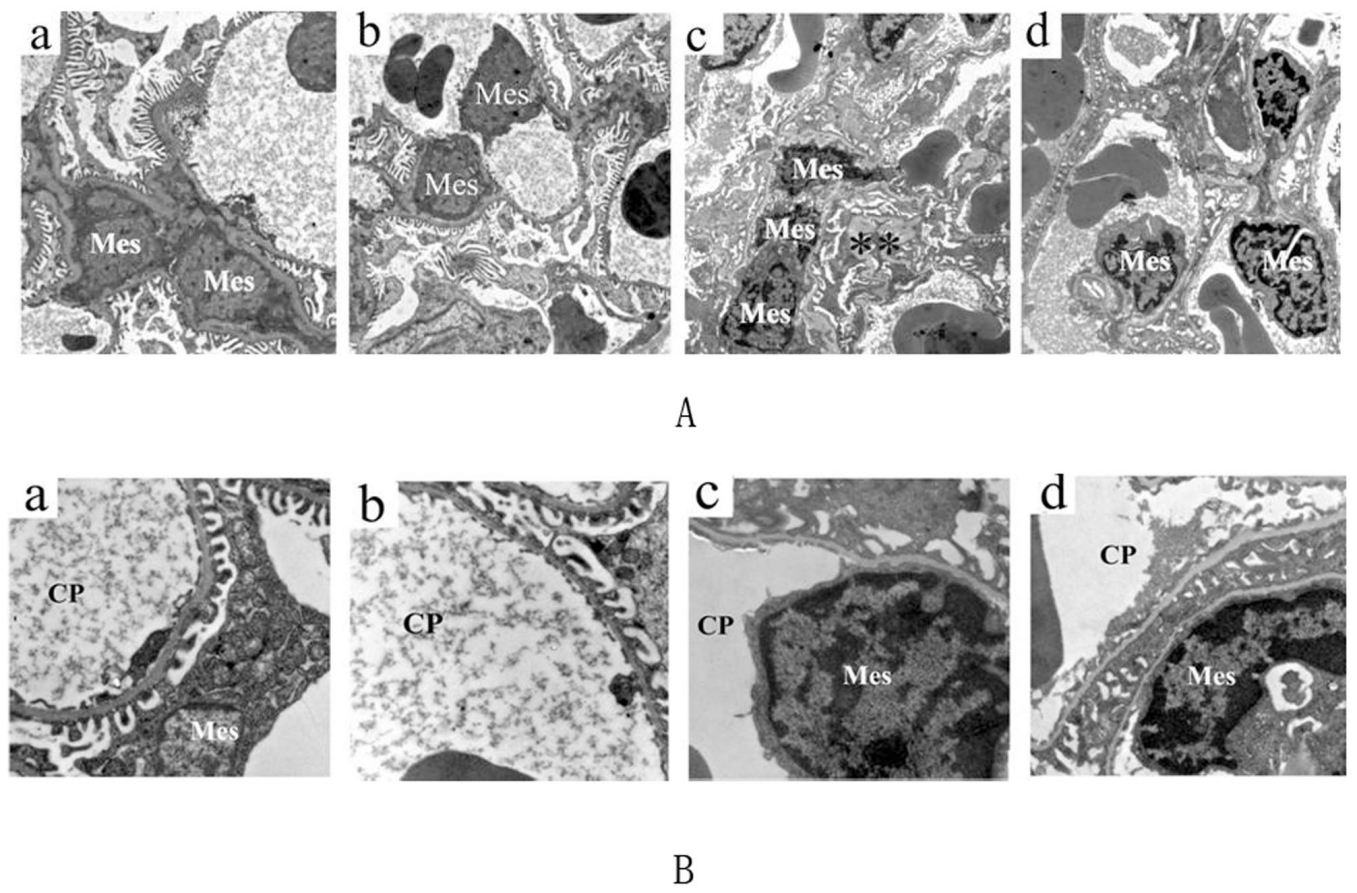
Effects of BBR on glomerular ultrastructural structure. **A**: a) EM analysis of a normal glomerulus revealed normal ultrastructure of the mesangial area. b) Almost normal ultrastructure of the mesangial area in CTL+BBR rats. c) Proliferation of mesangial cell and matrix were evident in ARD rats, with mesangial matrix expansion indicated by an asterisk. d) The mesangial area morphology was almost normal after BBR treatment in ARD+BBR rats. Original magnification ×6000. **B**: a) EM analysis of a normal glomerulus showing normal ultrastructure of the foot processes. b) Foot process morphology was normal in CTL+BBR rats. c) Focal areas of foot process effacement were evident in ARD rats. d) Foot process morphology was normal after BBR treatment in ARD+BBR rats. Original magnification ×15000. EM, electron micrograph; Mes, mesangial cell; CP, capillary lumen.

**Figure 4:**
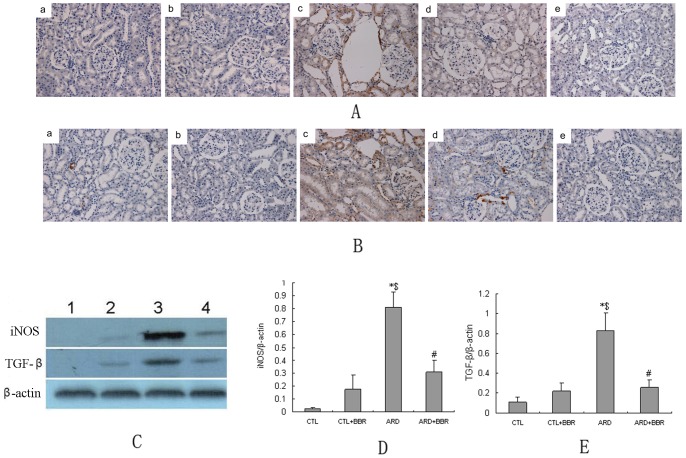
Effect of BBR on expression of pro-inflammatory and fibrotic molecules in rat kidney. **A** and **B**: Representative Immunohistochemical staining for iNOS and TGF-β, respectively. a) CTL rats had little staining for iNOS or TGF-β. b) Faint staining for iNOS or TGF-β in CTL+BBR kidneys. c) ARD caused stronger staining for iNOS or TGF-β in the renal endothelium and tubulointerstitium. d) BBR treatment caused a significant reduction in the expression of iNOS or TGF-β in ARD+BBR group kidney. e) Negative control. **C**) Expression of pro-inflammatory and fibrotic molecules in the kidney was detected by western blots; **D**–**E**: Each molecule densitometry analysis of western blots from 3 independent experiments. Magnification, ×200. Data expressed as mean ± SEM, n = 6 per group. ^*^ *P*<0.05 vs. normal group. ^#^ *P*<0.05 vs. ARD group. ^$^ *P*<0.05 vs. CTL+BBR

**Figure 5:**
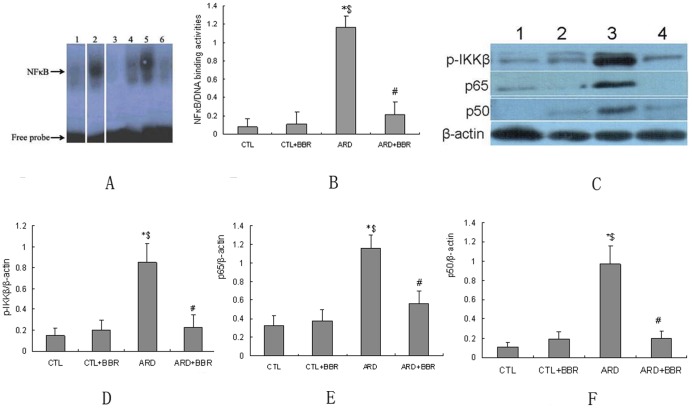
Effect of BBR on NFκB signaling pathway in rat kidney. **A**: Specificity and effect of BBR on NFκB-DNA binding activities in rat kidney. Lane 1: negative extract control; Lane 2: positive extract control; Lane 3: CTL group rats; Lane 4: CTL+BBR group rats; Lane 5: ARD group; Lane 6: BBR-treated rats. **B**: Densitometric analysis of NFκB-DNA binding activities. Densitometry was performed on autographs. Values were median relative density unit (RDU) of each group. **C**: Expression of phosphorylated-IKKβ, NFκB subunit p65/p50 in kidneys was detected by western blots.**D**–**E**: Each molecule densitometry analysis of western blots from 3 independent experiments. Data expressed as mean ± SEM, n = 6 per group. ^*^ *P*<0.05 versus CTL. ^#^ *P*<0.05 vs. ARD. n = 6 per group. *P<0.05 versus CTL group. ^#^ *P*<0.05 vs. ARD group. ^$^ *P*<0.05 vs. CTL+BBR

As shown in Tables 1, 2, 3 and Fig. 1, ARD rats exhibited increased SBP, accelerated renal blood flow, increased uALB, plasma cholesterol, LDL-cholesterol and TBARS, and decreased SOD activity as compared with CTL rats, indicating that ARD rats were characterized by renovascular stenosis, hypercholesterolemia, increased oxidative stress, impaired antioxidant capability, and albuminuria. Moreover, ARD rats had evident histological and even ultrastructural alterations, such as marked tubuloiterstitial inflammation and cell infiltration, tubular degeneration and ectasia, more interstitialperiglomerular, and periarterial fibosis, increased proliferation of mesangial cell and matrix, more focal areas of foot process effacement as examined by light microscope, electronic microscope (EM), and Western blotting analyses, respectively, as shown in Figs 2, 3, 4. In addition, the ARD rats displayed strong straining for iNOS and TGF-β in their renal endothelium and tubulointerstitium as measured by immunohistochemical staining as shown in Fig. 5. These data suggest that the ARD rat model is successful as anticipated.

**Table 1:** Changes in hypercholesterolemia and renal functional biomarkers in rats treated with or without BBR.

Groups	CTL	CTL+BBR	ARD	ARD+BBR
uALB (mg/24 h)	59±25	74±33#	409±43* ^$^	244±29* ^#$^
Cholesterol (mM)	2.14±0.49	2.75±0.53	4.77±0.86*	3.17±0.52#
Triglyceride (mM)	1.04±0.18	1.25±0.21	2.19±0.61	1.73±0.37
LDL (mM)	1.18±0.12	1.24±0.18#	2.58±0.19* ^$^	1.61±0.24#
sCr (µM)	69.9±15.4	66.7±16.2	102.6±34.7	58.1±22.3

Abbreviations used: CTL: Control, BBR: Berberine, ARD: Atherosclerotic renovascular disease. uALB: urinary albumin, LDL: low-density lipoprotein, sCr: serum creatinine.

Values were mean ± SEM, n = 6 per group.

* *P*<0.05 vs. CTL;

# *P*<0.05 vs. ARD;

$ *P*<0.05 vs.CTL+BBR

**Table 2:** Effects of BBR treatment on body weight and blood pressure in rats.

	Body weight (g)		SBP (mmHg)	
	Before BBR	After BBR	Before BBR	After BBR
CTL	447±16	509±18	139±6	136±4
CTL+BBR	451±13	514±13	132±4#	134±3#
ARD	476±25	536±27	179±11* ^$^	177±5* ^$^
ARD+BBR	478±21	504±19	181±7* ^$^	169±4* ^$^

Abbreviations used: CTL: Control, BBR: Berberine, ARD: Atherosclerotic renovascular disease, SBP: systolic blood pressure.

Values were mean ± SEM, n = 6 per group.

* P<0.05 vs. CTL;

# P<0.05 vs. ARD;

$ P<0.05 vs.CTL+BBR.

**Table 3:** Determination of pro-oxidants and antioxidants in rats treated with or without BBR.

	CTL	CTL+BBR	ARD	ARD+BBR
Total ROS (counts/mg protein/min)	856±326	877±334	1852±566	1201±412
SOD (U/mg protein)	63.58±3.21	64.12±5.24	23.00±5.52* ^$^	46.41±8.23#
TBARS (nmol/mg protein)	0.81±0.23	0.85±0.21	2.62±0.55* ^$^	1.01±0.32#
CAT (U/mg protein)	42.5±8.4	43.7±10.2	80.1±20.8	41.2±9.8

Abbreviations used: ROS: reactive oxygen species as measured with chemiluminescence; SOD: superoxide dismutase; TBARS: thiobarbituric acid reactive substances. CAT, catalase.

Values were mean ± SEM, n = 6 per group.

* P<0.05 vs CTL.

# P<0.05 vs ARD.

$ P<0.05 vs.CTL+BBR.

### Assessment of blood pressure and hemodynamics of renal artery

Of the all 24 coiled ARD rats, 16 (66.7%) were alive until Doppler examination, and 12 underwent further examination. In contrast, CTL rats undergoing sham operation all survived. Doppler ultrasound velocity at left renal artery was detected at 300 mm/sec in CTL groups. However, blood flow acceleration was detected at 600 mm/sec in ARD groups, indicating a 60% increase in renal artery stenosis as shown in Fig. 1. Compared with CTL, ARD rats had a significantly increased SBP (P<0.05), regardless of administration of BBR as shown in Table 2.

### Effects of BBR on serum lipid profiling and renal safety biomarkers in the ARD rats

Both uALB and serum creatinine (sCr), two of the eight qualified renal safety/functional biomarkers in rats, were assessed in this study. As shown in Table 1, ARD rats had a significant increase in cholesterol, LDL-cholesterol, and uALB as compared with CTL rats (P<0.05, respectively), and also had a marked increase in triglyceride and sCr, which failed to reach statistical significance. However, 12-week BBR treatment in ARD rats resulted in significantly decreased LDL-cholesterol and uALB (P<0.05, respectively), and markedly decreased cholesterol, triglyceride and sCr without reaching statistical significance after Bonferroni correction for the multiple comparisons (P>0.05).

### Effects of BBR on histological and ultrastructural alerations in the ARD rats

HE- and Masson trichrome- staining of kidney tissue revealed that ARD kidney exhibited more severe structural damage than CTL kidney, characterized by sclerotic glomeruli, more extensive regions of focal interstitial fibrosis and tubular atrophy with lymphocytic infiltrates as shown in Fig. 2A–B. Morphological evaluation of tubular damage (Fig. 2C) supported these results. EM studies identified focal effacement of foot processes, proliferation of mesangial cells and matrix production, consistent with the findings assessed by light microscopy as shown in Fig. 3. Furthermore, compared with ARD rats, BBR-treated rats exhibited less pronounced matrix formation, tubular damage and interstitial fibrosis (Figs. 2 and 3).

### Effects of BBR on oxidative stress status in the kidney

Oxidative stress biomarkers were assessed by measuring total ROS, SOD, TBARS and CAT in kidney tissues. There was a significant decrease in TBARS levels in response to 12-week BBR treatment in ADR rats (P<0.05) as shown in Table 3, implying decreased production of lipid peroxidation by BBR. A marked decrease in total ROS and CAT and a pronounced increase in SOD activity were seen with 12-week BBR treatment, but failed to reach statistical significance after Bonferroni correction for the multiple comparisons. These data indicated that BBR treatment reversed attenuated SOD activity, and that BBR could improve SOD-mediated superoxide anion scavenging.

### Effect of BBR on the expression of pro-inflammatory and fibrotic molecules

The expression of pro-inflammatory molecule iNOS was markedly elevated in the glomerular and tubular compartments, and profibrotic factor TGF-β was mainly distributed in the glomerular, perivascular, and tubulointerstitium of ARD kidneys, compared with CTL rats. That increased expression was substantially inhibited in the BBR-treated rats (Fig. 4), indicating an attenuation in renal inflammation.

### Effect of BBR on NFκB signaling pathway

NFκB-DNA binding activities were measured using EMSA. ARD rats had significantly increased NFκB activities as compared with CTL rats, and that increase was associated with increased expression of phosphorylated IKKβ, NFκB subunit p65/p50 and was reversed by BBR treatment (Figs. 4c, 5). The above evidence suggested that the mechanism underlying the suppression of both intrarenal inflammation and tubulointerstitial injury by BBR is involved in the down-regulation of NFκB signaling pathway.

## Discussion

In this study, the ARD rats exhibited renal dysfunction, increased renal oxidative stress and inflammation as well as tubulointerstitial fibrosis, along with impaired renal structure as compared with the CTL rats. The major findings were summarized as follows: 1) BBR treatment protected against worse renal function and structure in the ARD rats in addition to a significant reduction in hypercholesterolemia; 2) BBR suppressed renal oxidative stress and inflammation, improved renal antioxidant defense capacity, and modulated the expression of iNOS and TGF-β in the kidney; and 3) BBR attenuated elevated NFκB-DNA binding activity and protein levels of subunits p65/p50 and IKKβ.

Emerging evidence has demonstrated that BBR has multiple beneficial effects, such as lipid-lowering, hypoglycemic, insulin-sensitizing, and weight-lowering properties in diabetes and cardiovascular diseases [5], [9], [16]–[18]. However, renoprotective effect of BBR and its molecular mechanisms in ARD and chronic kidney injury remains to be determined.

Oxidative stress, as a result of the excessive production of free radical species, is one of the clinical characteristics in patients with hypertension and atherosclerosis [19]–[20]. Conversely, hypertension and atherosclerosis have been shown to cause oxidative stress in the kidney [21]. This self-perpetuating cycle can lead to progressive renal disease. Experimental blockade of the oxidative stress pathway with antioxidant vitamins in several disease models has been shown to decrease renal injury [19]–[21]. In the case of renal oxidative stress during atherosclerotic renovascular kidney injury, the predominant component of free radical is superoxide, which results in decreased SOD levels in the ARD rats. That highlights the point that oxidative stress caused by overproduction of oxidants and impaired antioxidant defense system occurred in the ARD rats may be responsible for observed renal damage in these rats. A previous study indicated that BBR possesses anti-oxidative effects via decreasing NADPH oxidase-dependent ROS production in vitro [22]. The results of our work also demonstrated that antioxidant activity and the scanverge rate of total ROS and superoxide were significantly higher in the ARD rats treated with BBR than in those without. These results suggest that BBR protects against oxidative renal damage by attenuating free radical production and preserving SOD and CAT activities, thereby improving BP, renal structure and function [23]–[24].

Proinflammatory cytokines involved in renal oxidative stress can activate the redox-sensitive transcription factor, NFκB, which, naturally occurring in its heterodimer state of the Rel protein family subunits p65/p50, can increase the production of ROS and reactive nitrogen species, such as superoxide and peroxynitrite [12], [25]–[27]. These ROS themselves can also increase NFκB activity, leading to further oxidative/nitrosative insult, which perpetuates this vicious positive feedback cycle and accelerates renal damage [28]. Once activated, NFκB heterodimers p65/p50 binds to NFκB binding sites on the promoter regions of its target genes to initiate the transcription and protein expression, such as iNOS and TGF-β, leading to significant inflammatory responses [29]–[31]. Activation of NFκB is dependent upon the activation of IKK2 (also known as IKKβ) through phosphorylation of the IκB molecule that is the inhibitor of NFκB. The kinase activity of IKKβ targets two adjacent serine residues of IκB, leading to its ubiquitination and proteasomal degradation, and release and activation of NFκB [31]–[32]. Many signaling pathways can activate NFκB converge at the level of IKKβ. Examples of stimuli leading to IKKβ and subsequent NFκB activation include inflammatory cytokines, endotoxins, viral infection, and ROS [30], [31], [33]–[35].

In this study, we observed that impaired renal function in ARD rats is the result of increased oxidative stress, pro-inflammation and tubulointerstitial fibrosis, consistent with the results of earlier observations [11]–[12]. As the rate-limiting enzyme, iNOS catalyzes the synthesis of NO from the guanidino nitrogen of L-arginine, resulting in the excessive production of NO, which involves in glomerular mesangial expansion, capillarectasia and tubulointerstitial fibrosis during the chronic renal damage [30], [31], [33]–[35]. In addition, TGF-β may mediate renal fibrotic injury through activation of the TGF-β/Smad pathway to facilitate extracellular matrix accumulation [29]. BBR treatment resulted in attenuated levels of intrarenal pro-inflammation and fibrosis molecules, suggesting that BBR protects against kidney injury through its anti-inflammation and anti-fibrotic effects. In this work, we observed for the first time that BBR can effectively inhibit NFκB activity and decrease the expression of phospho-IKKβ, p65/p50, and their downstream elaboration of prototypical molecules iNOS and TGF-β in the ARD kidneys, suggesting that BBR ameliorates intrarenal inflammation and tubulointerstitial injury, at least in part, through the suppression of NFκB signaling pathway.

In summary, we conclude that BBR intervention for the ARD rats can suppress proinflammatory and profibrosis responses, improve redox status in the kidney, lower hypercholesterolemia, and eventually ameliorate renal injury, and that such effects appear to be mediated by inhibition of the activity of the NFκB signaling pathway. Thus, BBR might play an important role in delaying progression of chronic kidney injury through preserving renal structure and function in patients with ARD.
